# Physicians’ Manual of Therapeutics

**Published:** 1901-01

**Authors:** 


Physicians' Manual of Therapeutics.—Parke, Davis & Co. have just issued this book, and write us to say that they wish the readers of the *Texas Medical Journal* to know that a copy will be sent free, prepaid, to the address of any physician who will drop them a line, asking for it,—mentioning the *Journal,* of course. This is no cheap stuff, but an *elegant volume* of 500 pages, bound in costly *flexible morocco.* It has been prepared with great care and skill, and containing as it does, a "complete presentation of available forms of every current remedy," and much other information, it will be found exceptionally useful as a work for ready and frequent reference. It can be carried in overcoat pocket, being only $4\frac{1}{2}$x7x1.

The Governor in his message to the Texas Legislature (Jan. 11th, inst.) made no recommendation or suggestion as to a State Board of Health,—nor any sanitary legislation whatever. He had the following paragraph, however, in his message, relative to the practice of medicine:

THE PRACTICE OF MEDICINE.

Immediate legislation is urgently needed touching the practice of medicine and surgery in this State.

The life and health of the people is being constantly endangered through the ignorance of many who under our laws are permitted to follow the profession. Certificates and diplomas have been openly sold for the purpose to all who would buy them, and permission to practice has been rarely withheld, however unprepared the applicant might have been to successfully pass the proper examination.

This information comes from every section of the State, and the evil has been for some time an object of severe denunciation by the press.

